# Beyond BMI: Exploring Adolescent Lifestyle and Health Behaviours in Transylvania, Romania

**DOI:** 10.3390/nu17020268

**Published:** 2025-01-13

**Authors:** Alexandra-Ioana Roșioară, Bogdana Adriana Năsui, Nina Ciuciuc, Dana Manuela Sîrbu, Daniela Curșeu, Ștefan Cristian Vesa, Codruța Alina Popescu, Andreea Bleza, Monica Popa

**Affiliations:** 1 Department of Community Medicine, “Iuliu Hatieganu” University of Medicine and Pharmacy, 400349 Cluj-Napoca, Romania; alexandra.rosioara@umfcluj.ro (A.-I.R.); nina.ciuciuc@umfcluj.ro (N.C.); dsirbu@umfcluj.ro (D.M.S.); dcurseu@umfcluj.ro (D.C.); andreea.bleza@elearn.umfcluj.ro (A.B.); monica.popa@umfcluj.ro (M.P.); 2 Research Center in Preventive Medicine, Health Promotion and Sustainable Development, “Iuliu Hatieganu” University of Medicine and Pharmacy, 400349 Cluj-Napoca, Romania; 3 Department of Pharmacology, “Iuliu Hatieganu” University of Medicine and Pharmacy, No. 23 Marinescu Street, 400337 Cluj-Napoca, Romania; stefan.vesa@umfcluj.ro; 4 Department of Abilities Human Sciences, “Iuliu Hatieganu” University of Medicine and Pharmacy, 400012 Cluj-Napoca, Romania; cpopescu@umfcluj.ro

**Keywords:** adolescent health, schools, risky behaviors, protective factors, nutritional status, screen time, physical activity, diet, lifestyle choices, hygiene

## Abstract

Background/Objectives: This study aimed to investigate the lifestyle and the behavioral factors that influence the nutritional status of adolescents from Transylvania, Romania. Methods: The Global School-Based Student Health Survey (GSHS) was used to collect data from 900 adolescents between 11 and 18 years old from the Transylvania region, Romania. This study assessed nutritional status by calculating BMI indicators adjusted to Z-Score, cut-off points according to the World Health Organization (WHO), using self-reported weight and height; perceived health status; food vulnerability; physical activity; addictive behaviors (cigarette, alcohol and drug consumption); number of hours spent in front of the computer/phone; hand and oral hygiene; sitting time/day; and sleep. Multivariate logistic regression was used to establish the lifestyle factors that influenced nutritional status. Results: The results showed that 8.7% (*n* = 78) of girls and 15.2% (*n* = 137) boys were overweight and obese. In total, 75% of the respondents were engaged in sedentary behaviors, and 65.8% (*n* = 592) had more than 2 h/day of screen exposure, considering that 98.7% of the study population had a mobile phone. The Romanian adolescents had poor dietary behaviors: over 80% of them did not meet the recommended amount of vegetable and fruit intake per day. Increased BMI was associated with higher-strength physical exercise and with being a boy. Conclusions: While some positive trends are evident, such as good oral and hand hygiene and low prevalence of smoking and drug use, significant challenges remain in areas like nutrition, physical activity, alcohol consumption and screen time.

## 1. Introduction

Adolescence (ages 10–19), a period of significant developmental changes, presents health risks often overlooked due to the perception of adolescents as generally healthy [1]. These risks, including physical inactivity, unhealthy eating habits and substance abuse, can have long-term health consequences extending into adulthood [2].

The rise in non-communicable diseases (NCDs) poses a significant challenge to achieving universal health coverage [3]. Unhealthy dietary habits and physical inactivity are key drivers of the rising NCD burden in developing countries [4,5].

Many risk factors for NCDs are preventable. Healthy lifestyles across all life stages, including pre-conception, childhood and adolescence, are crucial for mitigating the prevalence of non-communicable diseases (NCDs) [6,7,8,9,10,11]. Educating youth on NCDs and their risk factors is essential, as childhood behaviors significantly impact adult health outcomes [12]. Furthermore, personalized interventions can effectively improve dietary habits in this population [13].

Romania faces significant obstacles to providing high-quality healthcare to children and young people [14]. The prevalence of dental caries is high throughout children and teenagers. In terms of eating habits and the nutritional status of adolescents, a worrying proportion of them are overweight or obese, which can lead to a variety of future physical and psychological problems. In terms of physical activity, few adolescents demonstrate regular fitness practices. Romania presents an higher level of alcohol and tobacco consumption among this age sector [14]. Romania continues to have the EU’s lowest health expenditure as a share of gross domestic product (GDP) and per inhabitant [15].

The rising global consumption of sugar-laden soft drinks [16,17,18,19,20], with adolescent prevalence ranging from 3.3% to 80% across 107 countries [16], raises concerns among health professionals and policymakers. In Romania, there is a tendency of increasing consumption of sweetened drinks in young people [21,22].

A meta-analysis of Global School-Based Student Health Surveys, which included 94 countries screened in 72 studies regarding fruit and vegetable consumption, showed that overall, 34.5% consumed fruit less than once per day, 20.6% consumed vegetables less than once per day and 42.8% consumed carbonated soft drinks at least once per day [23].

In Romania, many adolescents do not meet [21] the recommended amount of daily intake of fruits and vegetables [24]. Consuming over 400 g of fruit and vegetable (FAV) daily is linked to increased vitamin intake and higher blood vitamin concentrations, particularly antioxidant and B vitamins [25,26], and a low level of fruit intake is associated with anxiety-induced sleep disturbance and suicidal ideation [27]. In Europe, many children and adolescents do not meet recommended intakes for plant-based foods, including fruits, vegetables, legumes and whole-grain cereals [28].

Adolescence offers a critical window to shape dietary habits for immediate and life-long health, yet the global understanding of adolescent diets remains limited [19,29].

Food insecurity (FI) is a critical socioeconomic and public health problem globally, particularly affecting children’s nutritional status and development, and a higher severity of FI is positively associated with a lower intake of iron [30], so global action is urgently required to address food insecurity among adolescents [30]. According to the World Bank 2021 reports, Romania had score of 16.3% of moderate food insecurity in its population [31]. In the 2024 Global Hunger Index, Romania is one of 22 countries with a GHI score of less than 5, which means a level of hunger that is low [32].

The World Health Organization (WHO) and other national health organizations recommend at least 60 min of daily moderate-to-vigorous physical activity for adolescents to maintain health [33,34]. Many studies prove that many adolescents do not meet this recommendation and engage in sedentary behavior all over the world [35,36], although school interventions seem to have some effects, better seen in normal-weight or overweight children [37]. Also, unhealthy behaviors such as excessive screen time are common among young Romanian people [21].

Adolescent health in Romanian schools is reflected in their diverse health behaviors and hygiene practices, which are influenced by factors such as education, environment and personal habits [14]. Poor personal hygiene increases the risk of illness, yet the prevalence of hygiene practices among adolescents is insufficiently described in Romania. According to our knowledge, there are no mandatory standardized programs in our schools implementing health education including hand hygiene or oral hygiene; instead, this is a flexible curriculum program for schools to implement. Even though there is a law of healthcare that passed in 2022, which includes health education in schools [38], it still does not have application methods or guidelines.

Regarding addictive behaviors in Romania, adolescent tobacco consumption remains high (lifetime consumption: 49.5%), with similar rates for boys and girls [39,40]. In Romania, per capita alcohol consumption exceeds European averages for boys in the 15 to 19 and 20 to 24 age groups. Girls in the 20 to 24 age categories also surpass the European average [39]. The most consumed illicit drug among teenagers in our country continues to be cannabis/hashish [39,41].

This study aims to provide novel insights into the relationship between lifestyle, behavior and nutritional status among adolescents in Transylvania, Romania, reported on gender differences, using data collected with Global School-based Student Health Surveys (GSHS).

The purpose of the GSHS is to provide data on health behaviors and protective factors among adolescents to help countries set priorities, develop programs and advocate for resources dedicated to school health programs and youth health policies and prevention programs targeting specific risk factors and groups of adolescents who may be more vulnerable [11].

## 2. Materials and Methods

### 2.1. Study Design and Population

We performed a cross-sectional study, and it was conducted based on a questionnaire adapted from a standardized one, distributed both online and physically among middle and high school students in Romania. A total of 900 responders were included in the study, between 11 and 18 years of age.

The Global School-Based Student Health Survey (GSHS) [11] was used, which is a school-based survey conducted primarily among students aged 13 to 18, which was developed by the World Health Organization (WHO) and the Centers for Disease Control and Prevention (CDC), in collaboration with UNICEF, UNESCO and UNAIDS.

Romania has a population of 2.132.738 11- to 18-year-olds [42]. Out of this total, 72% are enrolled in formal education, in 5th to 12th grades, which corresponds to our target age group. Of them, 72% (*n* = 1.318.298) are in school [43]. We only sampled schools from the public sector, this being the most representative form of study (97.7% of all children enrolled in school are learning in the public sector) [44].

We calculated the representative sample size for our study using Paniott’s formula, with a confidence level of 98% and a margin of error interval of 4%. We received a total of 848 responders.

Inclusion criteria for responders: school-going, gymnasium students, high school students, aged 11–18 years.

Exclusion criteria for responders: not going to school, outside the age range 11–18 years old, refusal of parents to sign the parental consent form, incomplete questionnaires.

**Figure 1:**
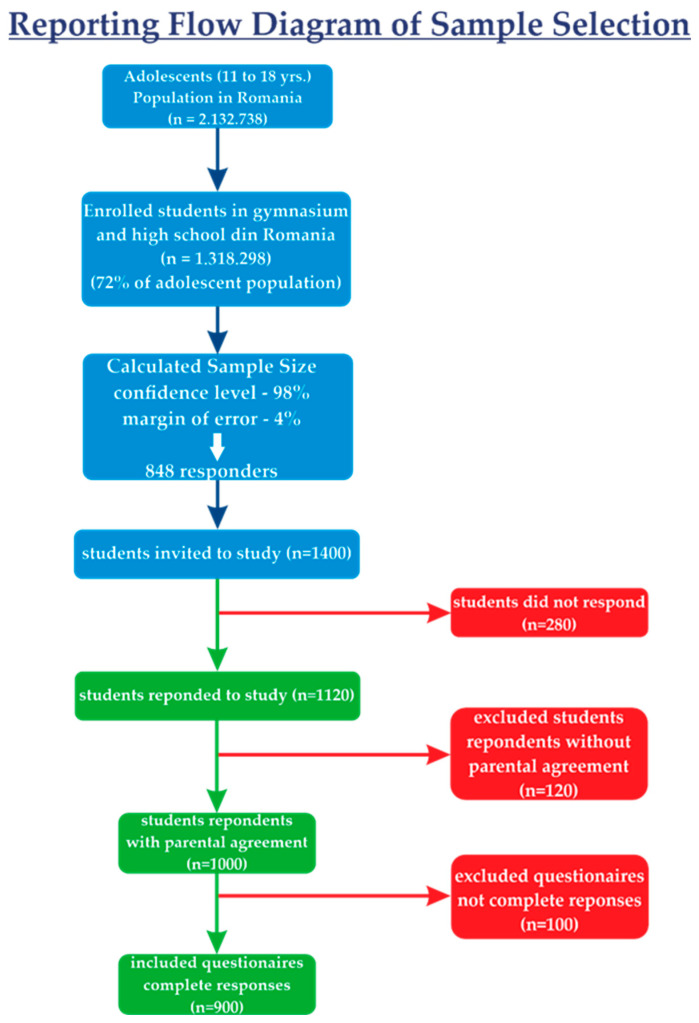
Reporting flow diagram of sample selection in the study.

Figure 1 showcases the reporting flow diagram of the sample of the study.

### 2.2. Data Collection and Questionnaire Measurements

The GSHS core questionnaire evaluates the following ten modules: “Alcohol use; Dietary behaviors; Drug use; Hygiene; Mental health; Physical activity; Protective factors; Sexual behaviors that contribute to HIV infection, other sexually transmitted infections and unintended pregnancy; Tobacco use; Violence and unintentional injury” [11].

In the present study, six basic modules were selected, including alcohol use (4 questions), dietary behavior (7 questions), physical activity (6 questions), smoking (6 questions), drug use (4 questions) and hygiene (6 questions), in order to assess the factors that are influencing the nutrition and the physical activity of the adolescents. Note that it is recommended that countries choose at least six of the core modules [11].

The students’ Body Mass Index (BMI) values were collected using self-reported weight and height. We calculated BMI = Weight (kg)/Height^2^ (m^2^). Subjects from the sample were categorized as underweight, normal weight, overweight and obese according to World Health Organization (WHO) cut-off points [45].

The questionnaire was completed voluntarily and anonymously during the school year 2023–2024 (September 2023–June 2024), and it included a sample of 900 students from different schools, both in rural and urban areas, from the larger part of Romania, (North-West regions). All the questionnaires that were incomplete and without parental consent were excluded.

To gain access to schools, a signed agreement from school management was required, followed by the distribution of questionnaires in schools. For the online version, a parental consent form accompanied by a QR code linking to the online questionnaire was printed. After the parents signed the consent form, the children scanned the QR code and completed the form. For the face-to-face version, after the school management signed the entry agreement, the printed questionnaire, which included the consent form, was distributed. The children took it home, had their parents sign it and then completed it themselves, after which the questionnaires were collected. The questionnaire took approximately 20 min to complete.

To validate it internally, the questionnaire was translated into Romanian and retranslated to English by an authorized translator, to be sure that the original meaning of the questions was kept; for reliability, it was pretested on 30 adolescents in the same age range. We performed linguistic validation, and we measured internal consistency on a pilot sample; Cronbach’s Alpha was 0.75.

In Table 1, the questions and coding for variables to be included are presented.

### 2.3. Ethical Considerations

This study was conducted according to the guidelines of the Declaration of Helsinki and approved by The Ethics Committee of the Cluj Napoca University of Medicine and Pharmacy (No. 179/20 September 2024). Also, the school’s management provided a signed agreement for collecting data, and all the parents of the adolescents included in the study signed an informed consent form.

### 2.4. Statistical Analyses

All statistical analyses were conducted using IBM SPSS Statistics (version 21, IBM Corp., Armonk, NY, USA) and Microsoft Excel (Microsoft Office 2010, Albuquerque, NM, USA). Descriptive and inferential analyses were performed to address the study’s research questions regarding adolescent health behaviors and their determinants.

Continuous variables (age and BMI) were summarized using means, standard deviations and 95% confidence intervals. Categorical variables (gender, physical activity levels and screen time) were described using frequencies and percentages. To explore differences in health behaviors and outcomes between boys and girls, chi-square tests were employed for categorical variables (BMI categories, physical activity levels and hygiene practices). The relationships between key lifestyle factors (e.g., screen time, physical activity and dietary habits) and nutritional outcomes (BMI classification) were tested using chi-square tests and cross-tabulations to identify statistically significant associations.

To identify independent predictors of adolescents’ nutritional status, a multivariate logistic regression model was employed. The dependent variable was nutritional status (normal weight vs. overweight/obese), and the variables that achieved significance in the univariate analysis included gender, physical exercise frequency, screen time per day, daily sleep duration and parental rules for screen time.

Statistical significance was set at *p* < 0.05 for all tests.

## 3. Results

### 3.1. Demographic Characteristics of the Study Group

The final sample size was 900 students from Transylvania, Romania, enrolled in public schools in grades 5 to 12, aged between 11 and 18 years old. The mean (±SD) age of the study population was 15.5 ± 1.92. Regarding their background, 639 (71%) were from urban areas, and 261 (29%) were from rural areas. Out of the total number of responders to this questionnaire, 474 (52.7%) were girls, and 426 (47.3%) were boys.

### 3.2. Assessment of Nutritional Status and Health Status

**Figure 2:**
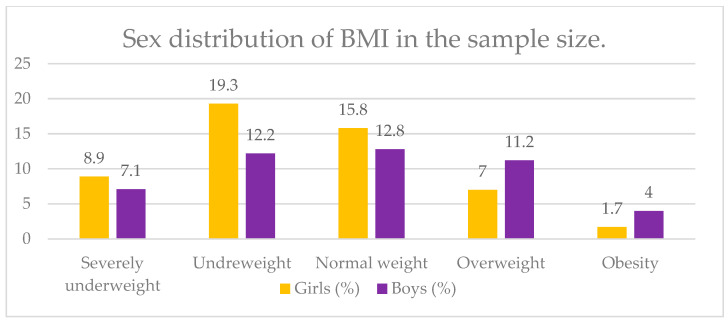
Sex distribution of BMI in the sample size.

We calculated the BMI of the adolescents and stratified the sample depending on the classes of obesity with WHO cut-off points for adolescents—severely underweight, underweight, normal weight, overweight and obese [45]. The study results showed that 28.2% (*n* = 254) of girls were underweight, and only 8.7% were overweight or obese. Regarding the boys, almost 20% were underweight (*n* = 174), and only 15.2% corresponded to the overweight or obese class; the rest of them had a normal weight (Figure 2).

This study conducted a descriptive analysis regarding perceived health status and food vulnerability. As shown in Table 2, 86.4% of all respondents had a positive perception about their health, meaning that they perceived their health as good or very good [46]. Regarding food vulnerability, 84.7% did not experience food insecurity, and for those who did, almost all responded “rarely” to the question “During the past 30 days, how often did you go hungry because there was not enough food in your home?”.

### 3.3. Dietary Behaviors

This study evaluated the dietary behaviors of the adolescents, and as seen in Table 3, the results show that, for the vast majority of the respondents from both groups, the recommended daily intake was not met [47], showing that a total of 88.2% of the responders did not consume at least three portions of vegetables per day and 80.6% did not consume at least two portions of fruit per day.

The consumption of carbonated and sugary drinks was considered an unhealthy dietary behavior. For both types of products, almost 70% of the responder’s daily intake was less than one drink per day.

### 3.4. Addictive Behaviors—Alcohol, Cigarette and Drug Consumption

When discussing addictive behaviors, we asked the adolescents about smoking, drinking alcohol and drug consumption.

This study revealed a relatively low prevalence of smoking behaviors among Romanian adolescents. (Table 4). The majority of both girls (59.7%) and boys (62.4%) reported never having smoked. Among those who had smoked in the past 30 days, 19.2% were girls and 17.6% were boys. Electronic cigarette use was slightly lower, with 16.7% of girls and 12.2% of boys reporting recent use.

This study investigated alcohol consumption among Romanian adolescents. Over half (61.6%) reported lifetime alcohol consumption, with no significant difference between genders (*p* = 0.047). However, a significant gender disparity emerged regarding age of initiation. While 43.6% reported never having more than a few sips of alcohol, 28.6% of boys versus 16.5% of girls initiated consumption before age 14 (*p* < 0.001). Sources of alcohol included family (8.2%), friends (12.2%) and other sources like shops or church events (14.1%), despite legal restrictions (*p* < 0.001). Worryingly, recent alcohol consumption (past 30 days) was reported by 28.9% of girls and 37.8% of boys (*p* = 0.005).

Regarding drug consumption, only 2.3% of all responders admitted that they tried drugs during their life (*p* = 0.008), with 1.7% saying that they tried cannabis and 0.6% saying that they tried amphetamine or methamphetamine, with a percentage of 0.3% starting before 14 years of age.

### 3.5. Physical Activity and Sedentary Behaviors

Analysis of physical activity levels revealed concerning trends (Table 5). Over half of the adolescents did not meet international recommendations [34,48]: 54.8% did not engage in at least 60 min of daily physical activity (*p* < 0.001), 52.8% performed insufficient exercise (*p* < 0.001) and 57.3% did not participate in physical education classes (*p* = 0.003).

Sedentary behaviors were prevalent, with 75% reporting excessive sitting time. Furthermore, insufficient sleep (<8 h/night) was reported by 61.6% of girls and 56.3% of boys.

### 3.6. Hours Spent in Front of the Computer (Screen Time) and Social Networks

This study examined social media use, screen time and parental rules among adolescents. A majority (68%) used social networks for over 2 h per day, with a significant gender difference: 73.2% of girls versus 62.2% of boys exceeded this threshold (*p* < 0.001).

Overall, 65.8% of participants reported more than 2 h of daily screen time (*p* = 0.035), with girls again more likely to have higher screen exposure. Alarmingly, some adolescents reported exceeding 8 h of daily screen time.

Despite these trends, 69.6% of adolescents reported a lack of parental rules governing screen time and social media use. This absence of regulation was consistent across genders (*p* = 0.562). Access to technology was widespread, with 98.7% of adolescents owning a personal mobile phone (Table 6).

### 3.7. Hygiene—Oral Health and Washing Hands

The study also analyzed the hygiene of the adolescents, both oral hygiene habits and hand hygiene habits (Table 7).

The results show that most adolescents (78%) cleaned their teeth twice a day, with a significant gender difference between girls and boys (*p* < 0.001), with girls being much more likely to clean their teeth twice a day (86.9% vs. 68.1%). The majority of adolescents (65.8%) did not use fluoride toothpaste, or they did not know if they used one, with no statistical difference between sex groups. Only a small percentage (3.9%) of adolescents missed school due to gum problems. There was a significant difference between girls and boys (*p* = 0.05), with girls being more likely to have missed school due to gum problems (5.1% vs. 2.6%).

Most adolescents (97.3%) practiced hand hygiene before eating, with no significant gender difference (*p* = 0.15). Almost all adolescents (99.1%) practiced hand hygiene after using the toilet, with no significant gender difference (*p* = 1). The vast majority (98.9%) used soap when washing their hands. There was a significant difference between girls and boys (*p* = 0.037), with girls being more likely to use soap (99.6% vs. 98.1%).

### 3.8. Factors Associated with Nutritional Status

We ran a multiple regression to predict the nutritional status of adolescents, depending on sex, physical exercise, walking/cycling to school, sitting time per day, sleep hours and parental rules for screen time, as seen in Table 8. According to the results, boys had a higher probability of being overweight or obese than girls (*p* = 0.007). Nutrition status was also associated with a higher level of physical exercise (stretching) (*p* < 0.001).

## 4. Discussion

This study aims to provide novel insights into the relationship between lifestyle, behavior and nutritional status among adolescents in Transylvania, Romania, reported on gender differences, using data collected with the Global School-based Student Health Survey (GSHS).

Nutritional status was assessed in our study by the BMI indicator, adjusted to Z-score (WHO cut-offs), using self-reported weight and height. According to the results of our research, 8.7% (*n* = 78) of girls and 15.2% (*n* = 137) boys are overweight and obese. Data from the literature investigating trends in recent years of BMI and unhealthy dietary behaviors of children and adolescents in Romania show that the prevalence of overweight (including obesity) in children from the urban area of Western Romania was recorded at high levels, higher in boys and at pre-puberty ages [21,29,49,50,51,52]. Romania faces growing concern about children and adolescent obesity and interconnected health issues such as adult-onset hypertension and dyslipidaemia [14].

Regarding food vulnerability, our study shows that 15.5% (*n* = 138) of the respondents experienced this phenomenon, and of them, 11.2% (*n* = 101) responded that they rarely happened to not have food. According to the Global Food Security Index (2022), in the overall ranking table, Romania is in 45th position, with a score of 68.8 out of 100, after all the EU countries [53], and according to the Global Hunger Index (GHI) 2024, Romania is experiencing a low level of food insecurity [32]. Our results appear to be close to the report data of the World Bank (2021), where Romania had a score of 16.3% of moderate food insecurity in its population [31], but this is closer to the data reported last year by the GHI.

When investigating daily fruit and vegetable intake, our results highlighted that the vast majority of participants did not consume a proper quantity. In total, 80.6% of the responders ate fewer than two portions/day of fruit and 88.2% fewer than three portions/day of vegetables. Two other European studies have involved almost all countries—the WHO European Childhood Obesity Surveillance Initiative (COSI) [54,55], which investigated daily intake of fruit and vegetables in adolescents, showed that fruits are more popular than vegetables in the daily eating routine of adolescents, but the servings consumed were not sufficient [21]. Our results were consistent with previous studies investigating Romanian adolescents’ lifestyles [21,56]. It was observed that those adolescents who control the quality and quantity of their carbohydrates, by consuming more fruits and vegetables, were associated with increased physical activity, which reduced their cardiovascular risk [57] and asthma risk [58]. However, having even one serving per day of 100% fruit juice is associated with BMI gain among children [59].

This study’s findings on sugary and carbonated soft drink consumption contrast with previous research indicating increased intake among adolescents [19,21,22,60,61,62]. Contrary to expectations, the majority of participants in this study consumed less than one portion per day: 68.9% for carbonated drinks and 71% for sugary drinks, with 23% and 24%, respectively, reporting no consumption in the past week. This discrepancy may be attributed to several factors, including rising beverage prices [63,64] and decreased purchasing power in Romania, which is roughly half the European average [65]. Additionally, the socio-economic impact of regional conflict and inflation may play a role [66]. These findings suggest that economic constraints may be influencing adolescent consumption patterns, leading to a lower intake of sugary and carbonated drinks than previously observed.

This study examined substance use behaviours among Romanian adolescents, revealing some noteworthy trends. Regarding smoking, there was a relatively low prevalence overall, with the majority of both genders reporting never having smoked, and a higher prevalence of recent smoking among girls than boys. Electronic cigarette use was slightly lower than traditional cigarette smoking. A Danish study found that only 8.3% of Danish adolescents aged 15–17 were daily smokers [67]. A new WHO/HBSC (2021/2022) report reveals concerning trends in adolescent substance use [68], saying that E-cigarettes have surpassed conventional cigarettes in popularity, with 32% of 15-year-olds reporting e-cigarette use at some point and 20% in the past 30 days. On alcohol consumption, our results show that over half reported lifetime alcohol consumption, with no gender difference in prevalence with significant gender disparity in age of initiation, with boys more likely to start before age 14 and a high prevalence of recent alcohol consumption. Sources of alcohol were diverse, including family, friends and illicit means. This is consistent with the same recent WHO/HBSC report [68], which shows that alcohol is the most frequently consumed substance among adolescents, with 57% of 15-year-olds having tried alcohol at least once, and nearly 4 in 10 (37%) indicating that they consumed alcohol in the past 30 days. Regarding drugs, the results of this study showed a very low prevalence of lifetime drug use, with cannabis being the most commonly reported drug tried, and an extremely low prevalence of amphetamine or methamphetamine use. Cannabis being the most commonly reported drug is consistent with the most recent Romanian report on drug use [39] and with the WHO/HBSC [68] report on this category of age. A study analyzing trends in adolescent substance use [69], employing data from Estonian, Latvian, Lithuanian and Polish children 15 years of age, between 1994 and 2018, highlighted that the prevalence of cannabis use among boys fluctuated more (from 5% to 13%) than that among girls (from 2% to 8%).

Regarding oral health, this study found that 78% of adolescents reported brushing their teeth twice daily, and 96.1% had not experienced gum problems severe enough to cause school absence. However, these seemingly positive findings contrast with the existing Romanian literature [70,71,72,73,74,75,76], which documents a high prevalence of dental caries linked to sugary food and drink consumption, as well as varying levels of oral health knowledge and practices. Regarding hand hygiene, the present study shows that almost all participants practice hand hygiene before eating and after using toilets. Unfortunately, readily available, specific statistics on hand hygiene practices among Romanian adolescents are limited because of a lack of standardized programs in school. Another Romanian study shows improvement in that area of practices after targeted intervention [77].

This study revealed a concerningly high prevalence of inactivity among Romanian adolescents. Over half failed to meet international physical activity recommendations, including those related to exercise and physical education participation. Sedentary behaviors were widespread in both rural and urban environments, echoing findings from other Romanian and international studies [78,79,80,81,82].

Interestingly, a positive association was observed between nutritional status and stretching exercises, likely due to increased muscle mass. However, this study acknowledges the limitations of BMI as an indicator, as it does not differentiate between fat and fat-free mass.

Given the established link between sedentary behaviour and adverse cardiometabolic and aging outcomes in adults [80], these findings underscore the urgent need for increased efforts to promote physical activity and reduce sedentary time among adolescents. Educational interventions and public health initiatives targeting this population are crucial for fostering healthy lifestyle habits and mitigating future health risks.

This study found that 65.8% of Romanian adolescents exceeded 2 h of daily screen time, a finding consistent with other Romanian and European studies [21,83]. This prevalence of excessive screen time, likely exacerbated by the COVID-19 pandemic [84,85], warrants systematic attention due to its established negative impacts on adolescent physical and mental health. These findings emphasize the need for interventions and public health initiatives aimed at promoting healthy technology use and mitigating the risks associated with excessive screen time among young people.

This study acknowledges several limitations inherent to its cross-sectional design. Firstly, while associations between determinants and outcomes can be observed, this design can rule out the establishment of definitive causal relationships. Secondly, self-reported data on attitudes and behaviors may be susceptible to social desirability bias, potentially leading to an overestimation of positive health practices and an underreporting of less desirable behaviors, such as smoking and alcohol consumption. Thirdly, recall bias may have influenced the accuracy of the adolescents’ self-reported behaviors and health-related information. Fourth, this study’s geographical restriction to the Transylvania region limits the generalizability of findings to the broader population of Romanian adolescents. Fifth, the school-based data collection may have introduced the potential for peer influence on responses.

While acknowledging certain limitations, this study’s high response rate and geographically diverse sample offer compelling evidence regarding the impact of modifiable environmental factors, such as parental rules, school policies and community resources, on adolescent health, yielding important insights for promoting well-being across the lifespan.

The following are our suggestions for interventions and future research. Overall, our study clearly indicates that Romanian adolescents were engaged in sedentary behaviors, like excessive screen time and sitting time. More than half of the adolescents did not meet the international recommended level of physical activity and daily vegetable and fruit intake. Concrete interventions that can be suggested include promoting healthy eating through school-based programs promoting nutrition education and public awareness campaigns that target adolescents and their families with messages about the importance of balanced nutrition and the risks of unhealthy diets. To increase physical activity, schools should promote participation in extracurricular sports, dance classes and other physical activities and limit sedentary time, implementing strategies to reduce screen time and promoting breaks from sitting, both at school and at home. This implies the involvement of both schools and families. Future research should consider longitudinal studies, tracking changes in health behaviors and outcomes over time to assess the effectiveness of interventions and identify emerging trends and qualitative research to explore the underlying reasons for specific behaviors, such as the social and cultural factors influencing alcohol use or barriers to physical activity. By implementing these suggestions for interventions and future research, Romania can effectively address the health challenges faced by adolescents and promote their overall well-being.

## 5. Conclusions

This study provides a comprehensive overview of various health behaviors and indicators among Romanian adolescents. A proportion of adolescents, particularly girls, are underweight, highlighting the need to address potential nutritional deficiencies and promote healthy weight-gain strategies. Low fruit and vegetable consumption coupled with high levels of sedentary behavior and insufficient physical activity raise concerns about long-term health risks, including chronic diseases. The early initiation of alcohol consumption, especially among boys, and the high prevalence of recent alcohol use necessitate targeted interventions to address this risky behavior. This study highlights a complex picture of adolescent health in Romania. While some positive trends are evident, such as good oral and hand hygiene and low prevalence of smoking and drug use, significant challenges remain in areas like nutrition, physical activity, alcohol consumption and screen time. These findings underscore the need for comprehensive and targeted interventions to address these challenges and promote healthy lifestyles among Romanian adolescents.

## Figures and Tables

**Table 1:** Questions and coding for variables to be included in the analysis.

Variables	Questions	Coding
**Sociodemographic**		
Age	How old are you?	11 or younger, 12–14 “≤14” 15–18 or older “≥15”
Sex	What is your sex?	Female “Girls”, Male “Boys”
Background	What residential area do you live in?	“Rural”, “Urban”
**Well-being and Health**		
Food insecurity	During the past 30 days, how often did you go hungry because there was not enough food in your home?	Most of the time/Always Rarely/Sometimes “Yes” Never/ “No”
Self-perceived health status	How would you describe your health in general?	Excellent/Very good/Good “Positive Perception About Health” Acceptable/Poor “Negative Perception About Health”
Nutritional status (Calculated by BMI—WHO Z scores cut-off)	How tall are you without shoes? What is your weight?	<−2SD from median for BMI by age and sex “Underweight” >+1SD from median for BMI by age and sex “Overweight” >+2SD from median for BMI by age and sex “Obese”
**Nutritional Behaviors**		
Fruit daily intake	In the past 7 days, how many times did you eat fruits such as apples, pears, plums, bananas, melons, etc.?	Less than 2 portion/day (1/day/4 to 6 times in the last 7 days/1 to 3 times in the last 7 days/I haven’t eaten fruit in the last 7 days) “Yes—risk factor” 2 or more portion/ day “No—risk factor”
Vegetable daily intake	In the past 7 days, how many times did you eat vegetables such as tomatoes, cucumbers, carrots, lettuce, broccoli, etc. (excluding potatoes)?	Less than 3 portions/day (1/day/4 to 6 times in the last 7 days/ 1 to 3 times in the last 7 days/I haven’t eaten vegetables in the last 7 days) ”Yes—risk factor” 3 or more portions/day “No—risk factor”
Carbonated soft drinks	In the past 7 days, how many times did you drink a can, bottle or glass of a carbonated soft drink like Coca-Cola or Sprite?	More than 1/day “Yes—risk factor” Less than 1/day (4 to 6 times in the last 7 days/1 to 3 times in the last 7 days/I have not had any carbonated drinks in the last 7 days) “No—risk factor”
Sugary drinks	In the past 7 days, how many times did you drink a can, bottle or glass of a sugar-sweetened beverage (like sports drinks, energy drinks, sugar-sweetened teas, coffees, or flavoured waters)?	More than 1/day “Yes—risk factor” Less than 1/day (4 to 6 times in the last 7 days/1 to 3 times in the last 7 days/I have not had any sugary drinks in the last 7 days) ”No—risk factor”
**Physical Activity and Sedentary Behaviors**		
Physical activity	During the past 7 days, on how many days were you physically active for a total of at least 60 min per day? Add up all the time you spent doing any kind of physical activity each day.	0–4 days “Yes” “Insufficient Active” ≥5 days “No”—“Active enough”
Physical exercise—stretching	In the past 7 days, how many days did you do exercises to strengthen or tone your muscles, such as push-ups, squats, or weight lifting?	Less than 5 days/week (0, 1, 2, 3, 4)—“Insufficient Active” More than 5 days/week—“Active Enough”
Walking/cycling to school	In the past 7 days, how many days did you walk or bicycle to or from school?	0–2 days “Yes” “Insufficient Active” ≥3 days “No”—“Active enough”
Physical education class	In this school year, how many days did you attend the physical education class each week?	0–2 days “Yes” “Insufficient Active” ≥3 days “No”—“Active enough”
Sitting time hours/ day	On a typical day, how much time do you spend sitting and watching TV, playing computer games, chatting with friends, using your mobile phone?	≥3 h per day “Yes—risk factor” ≤2 h per day “No—risk factor”
Sleep hours per night	During school time, how many hours do you sleep each night?	<8 h per night “Not Enough Sleep” ≥8 h per night “Enough Sleep”
**Addictive behaviors influencing health.**		
Ever smoking	Have you ever tried smoking cigarettes, even a puff or two?	“Yes” “No”
Age beginning smoking	How old were you when you first tried smoking a cigarette?	I have never tried smoking a cigarette “Never” 7 years old or younger/8–14 years “≤14” 15–18 years “≥14”
Active smoking—normal cigarettes	In the past 30 days, how many days did you smoke cigarettes?	≥1 days “Yes” 0 day “No”
Active smoking—electronic cigarettes	In the past 30 days, how many days did you use electronic cigarettes?	≥1 days “Yes” 0 day “No”
Alcohol consumption	Have you ever consumed alcohol? (a serving is a glass of wine/beer/strength, etc.)	“Yes” “No”
Age of first alcohol consumption	How old were you when you first drank more than a few sips of alcohol?	I have never had a drink of alcohol other than a few sips “Never” 7 years old or younger/8–14 years “≤14” 15–18 years “≥14”
Active alcohol consumption	In the past 30 days, how many days did you drink at least one drink that contained alcohol?	≥1 days “Yes” 0 day “No”
How did they obtain the alcohol	In the past 30 days, how did you get the alcohol you drank?	I did not drink alcohol in the past 30 days “Never had alcohol” I got it from my family “Family” I gave someone else money to buy it for me/I got it from my friends “Friends” I bought it in a shop/I stole it or got it without permission/I got it in another way “Other sources”
Drug consumption	Have you ever used drugs? (e.g., cannabis, marijuana, amphetamines, cocaine, heroin)	“Yes” “No”
Age of first drug consumption (years)	How old were you when you first used drugs?	I have never used drugs “Never” 7 years old or younger/8–14 years “≤14” 15–18 years “≥14”
Cannabis consumption	In your lifetime, how many times have you used cannabis (also called marijuana or weed)?	≥1 days “Yes” 0 day “No”
Amphetamine and methamphetamine	During your lifetime, how many times have you used amphetamines or methamphetamines for non-medical purposes?	≥1 days “Yes” 0 day “No”
**Social behaviors—use of social networks**		
Screen time—hours/day	On a typical school day, how many hours of screen time do you spend?	Less than 2 h/day—“No risk factor” More than 2 h/day—“ Risk factor”
Use of social network	In the past 7 days, how many hours a day did you use your mobile phone for social networks, for online communication or to surf the Internet?	Less than 2 h/day—“No risk factor” More than 2 h/day—“ Risk factor”
Parental rules for screen time and social network	Do your parents or guardians have rules about how you can use social media, online communication or the Internet?	“Yes” “No”
Having a personal mobile phone	Do you have your own mobile phone to use?	“Yes” “No”
**Hand Hygiene and Oral Hygiene**		
Washing teeth 2/day	In the past 30 days, how many times a day did you usually brush your teeth?	I have not cleaned or brushed my teeth in the past 30 days/Less than once a day/Once a day “Not Properly” Twice a day/3 or more times a day “Properly”
Fluoride toothpaste	In the past 30 days, did you use a toothpaste that contains fluoride when you brushed your teeth?	I have not cleaned or brushed my teeth in the past 30 days/ No, I did not usually use a toothpaste that contains fluoride/I do not know if the toothpaste I usually used contains fluoride “No” Yes, I usually used a toothpaste that contains fluoride “Yes”
Gum problem → missing school	In the past 30 days, did you have any problems with your teeth or gums that caused you to miss school?	“Yes” “No”
Hand hygiene before eating	In the past 30 days, how often did you wash your hands before eating?	Most of the time/Always “Yes” Never/Rarely/Sometimes “No”
Hand hygiene after toilet	In the past 30 days, how often did you wash your hands after using the toilet or latrine?	Most of the time/Always “Yes” Never/Rarely/Sometimes “No”
Using soap while washing hands	In the past 30 days, how often did you use soap when washing your hands?	Most of the time/Always “Yes” Never/Rarely/Sometimes “No”

**Table 2:** Well-being and health—distribution between boys and girls (males and females).

Variable	Item	Total—*n* (%)	Girls—*n* (%)	Boys—*n* (%)	*p*-Value
Well-being and health					
Food/nutrition vulnerability	No	762 (84.7)	397 (83.8)	365 (85.7)	0.459
	Yes	138 (15.3)	77 (16.2)	61 (14.3)	
Perceived health status	Negative perception	122 (13.6)	89 (18.8)	33 (7.7)	<0.001
	Positive perception	778 (86.4)	385 (81.2)	393 (92.3)	
Nutritional status	Underweight, normal weight	685 (76.1)	396 (57.8)	289 (42.2)	<0.001
	Overweight, obese	215 (23.9)	78 (36.3)	137 (63.7)	

Chi-Square.

**Table 3:** Daily intake of fruits and vegetables, carbonated and sugary drinks.

Variable	Item	Total—*n* (%)	Girls—*n* (%)	Boys—*n* (%)	*p*-Value
Nutritional Behaviors					
Fruits daily intake	Less than 2 portion/day	725 (80.6)	384 (81)	341 (80)	0.736
	2 or more portion/day	175 (19.4)	90 (19)	85 (20)	
Vegetable daily intake	Less than 3 portion/day	794 (88.2)	426 (89.9)	368 (86.4)	0.12
	3 or more portion/day	106 (11.8)	48 (10.1)	58 (13.6)	
Carbonated soft drinks	more than 1/day	280 (31.1)	135 (28.5)	145 (34)	0.083
	less than 1/day	620 (68.9)	339 (71.5)	281 (66)	
Sugary drinks	more than 1/day	261 (29)	141 (29.7)	120 (28.2)	0.607
	less than 1/day	639 (71)	333 (70.3)	306 (71.8)	

Chi-Square.

**Table 4:** Addictive behaviors influencing health.

Variable	Item	Total—*n* (%)	Girls—*n* (%)	Boys—*n* (%)	*p*-Value
Addictive behaviors					
Ever smoking	Yes	351 (39)	191 (40.3)	160 (37.6)	0.412
	No	549 (61)	283 (59.7)	266 (62.4)	
Age beginning smoking	Never	554 (61.6)	287 (60.5)	267 (62.7)	0.226
	≤14	147 (16.3)	72 (15.2)	75 (17.6)	
	≥14	199 (22.1)	115 (24.3)	84 (19.7)	
Active smoking (last 30 days)—normal cigarettes	Yes	166 (18.4)	91 (19.2)	75 (17.6)	0.548
	No	734 (81.6)	383 (80.9)	351 (82.4)	
Active smoking (last 30 days)—electronic cigarettes	Yes	131 (14.6)	79 (16.7)	52 (12.2)	0.059
	No	769 (85.4)	395 (83.3)	374 (87.8)	
Alcohol consumption	Yes	554 (61.6)	277 (58.4)	277 (65)	0.047
	No	346 (38.4)	197 (41.6)	149 (35)	
Age of first alcohol consumption	Never	392 (43.6)	226 (47.7)	166 (39)	<0.001
	≤14	200 (22.2)	78 (16.5)	122 (28.6)	
	≥14	308 (34.2)	170 (35.9)	138 (32.4)	
Active alcohol consumption (last 30 days)	Yes	298 (33.1)	137 (28.9)	161 (37.8)	0.005
	No	602 (66.9)	337 (71.1)	265 (62.2)	
How did they obtain the alcohol	Never had alcohol	589 (65.4)	331 (69.8)	258 (60.6)	<0.001
	Family	74 (8.2)	34 (7.2)	40 (9.4)	
	Friends	110 (12.2)	70 (14.8)	40 (9.4)	
	Other sources	127 (14.1)	39 (8.2)	88 (20.6)	
Drug consumption	Yes	21 (2.3)	5 (1.1)	16 (3.8)	0.008
	No	879 (97.7)	469 (98.9)	410 (96.2)	
Age of first drug consumption (years)	Never	882 (98)	470 (99.2)	412 (96.7)	0.023
	≤14	3 (0.3)	0 (0)	3 (0.7)	
	≥14	15 (1.7)	4 (0.8)	11 (2.6)	
Cannabis consumption	Yes	15 (1.7)	2 (0.4)	13 (3.1)	0.003
	No	885 (98.3)	472 (99.6)	413 (96.9)	
Amphetamine and methamphetamine	Yes	6 (0.7)	470 (99.2)	424 (99.5)	0.689
	No	894 (99.3)	4 (0.8)	2 (0.5)	

Chi-Square.

**Table 5:** Level of physical activity and sedentary behaviors between study groups.

Variable	Item	Total—*n* (%)	Girls—*n* (%)	Boys—*n* (%)	*p*-Value
Physical activity—Sedentary behaviors					
Active 60 min/day	No	493 (54.8)	303 (63.9)	190 (44.6)	<0.001
	Yes	407 (45.2)	171 (36.1)	236 (55.4)	
Physical exercise—stretching	Less than 5 days/week	502 (55.8)	330 (69.6)	172 (40.4)	<0.001
	More than 5 days/week	398 (44.2)	144 (30.4)	254 (59.6)	
Walking/cycling to school	No	295 (32.8)	150 (31.6)	145 (34)	0.477
	Yes	605 (67.2)	324 (68.4)	281 (66)	
Physical education class	No	516 (57.3)	294 (62)	222 (52.1)	0.003
	Yes	384 (42.7)	180 (38)	204 (47.9)	
Sitting time hours/day	More than 2 h/day	675 (75)	368 (77.6)	307 (72.1)	0.064
	Less than 2 h/day	225 (25)	106 (22.4)	119 (27.9)	
Sleep hours per night	<8	532 (59.1)	292 (61.6)	240 (56.3)	0.118
	≥8	368 (40.9)	182 (38.4)	186 (43.7)	

Chi-Square.

**Table 6:** Social behaviors—use of social networks between boys and girls.

Variable	Item	Total—*n* (%)	Girls—*n* (%)	Boys—*n* (%)	*p*-Value
Social behaviors—Blue screens					
Screen time hours/day	Less than 2 h/day	308 (34.2)	147 (31)	161 (37.8)	0.035
	More than 2 h/day	592 (65.8)	327 (69)	265 (62.2)	
Use of social networks	Less than 2 h/day	288 (32)	127 (26.8)	161 (37.8)	<0.001
	More than 2 h/day	612 (68)	347 (73.2)	265 (62.2)	
Parental rules for screen time and social networks	Yes	274 (30.4)	140 (29.5)	134 (31.5)	0.562
	No	626 (69.6)	334 (70.5)	292 (68.5)	
Having personal mobile phone	Yes	888 (98.7)	470 (99.2)	418 (98.1)	0.246
	No	12 (1.3)	4 (0.8)	8 (1.9)	

*p* < 0.05 was considered statistically significant; Chi-Square.

**Table 7:** Hand hygiene and oral hygiene between boys and girls.

Variable	Item	Total—*n* (%)	Girls—*n* (%)	Boys—*n* (%)	*p*-Value
Hygiene Behaviors					
Cleaning teeth twice/day	No	198 (22)	62 (13.1)	136 (31.9)	<0.001
	Yes	702 (78)	412 (86.9)	290 (68.1)	
Fluoride toothpaste	No	592 (65.8)	314 (66.2)	278 (65.3)	0.779
	Yes	308 (34.2)	160 (33.8)	148 (34.7)	
Gum problem → missing school	No	865 (96.1)	450 (94.9)	451 (97.4)	0.05
	Yes	35 (3.9)	24 (5.1)	11 (2.6)	
Hand hygiene before eating	No	24 (2.7)	9 (1.9)	15 (3.5)	0.15
	Yes	876 (97.3)	465 (98.1)	411 (96.5)	
Hand hygiene after toilet	No	8 (0.9)	4 (0.8)	4 (0.9)	1
	Yes	892 (99.1)	470 (99.2)	422 (99.1)	
Using soap while washing hands	No	10 (1.1)	2 (0.4)	8 (1.9)	0.037
	Yes	890 (98.9)	472 (99.6)	418 (98.1)	

Chi-Square.

**Table 8:** Multiple regression regarding nutritional status and physical activity.

Model	B	S.E.	Wald	df	Sig.	Exp(B)	95% C.I. for EXP(B)	
							Lower	Upper
Sex	0.409	0.151	7.337	1	0.007	1.506	1.12	2.024
Physical exercise	1.526	0.152	101.235	1	<0.001	4.598	3.416	6.19
Walking/cycling to school	0.307	0.158	3.784	1	0.052	1.359	0.998	1.85
Sitting time hours/ day	0.23	0.179	1.647	1	0.199	1.258	0.886	1.787
Sleep hours per night	0.087	0.156	0.315	1	0.574	1.091	0.804	1.481
Parental rules for screen time	0.18	0.167	1.159	1	0.282	1.197	0.863	1.66
Constant	−0.072	0.092	0.623	1	0.43	0.93		

CI—confidence interval.

## Data Availability

The datasets generated and analyzed during the current study are not publicly available, since they were specifically collected by the authors for the present study, but may be available from the corresponding author on reasonable request.
